# From the Editor’s Desk

**Published:** 2008-11

**Authors:** Andries Brink

**Affiliations:** The Cardiovascular Journal of South Africa

The *Cardiovascular Journal of South Africa* (CVJSA) has made unprecedented strides in the past 18 months, based on steady progress since its inception in 1989.

In October 2007 the Journal became the *Cardiovascular Journal of South Africa* (CVJA), incorporating the CVJSA, in issue 5 of volume 18. It is now widely visible, not only in Africa, but also in North America, Europe and the Middle East.

Currently, both CVJSA and CVJA are recorded in databases by their printed and on-line ISSN numbers for indexing and listing purposes. These data can be retrieved and viewed, providing an integrated statistical history, some of which will be reflected for the month of September 2008 on page 308 in this issue.

Most importantly, the CVJA has now been accredited, indexed and abstracted in *Science Citation Index Expanded (SciSearch) and Journal Citation Reprints/Science Edition.* This brings the CVJA to the forefront of international medical literature and will enable the development of an impact factor. Although the impact factor for assessing quality of information leaves much to be desired, it is still the benchmark used by institutions and funders as a guide to decision making on career advancement and allocation of funds.

We make a special appeal to all authors and readers of this Journal to support the publication to their utmost, in order to increase the citation rate. CVJA data are also visible in the Elsevier competitor citation resource, known as Scopus.

The final issue of CVJA this year also marks the end of 20 years of production. Having come this far and achieved the above milestones, we have decided to celebrate the event early in 2009 with a celebratory historical issue, which has already been structured and is in the process of being compiled.

The sustained interest, contributions and support of the many readers, authors and reviewers in the field of cardiovascular medicine in South Africa and other countries have made this remarkable achievement possible. This is the only quality journal of its kind on the African continent. Thank you to all.

The celebratory events will also include a gala evening, where all are welcome. Please contact the Journal offices on cvjsa@cvjsa.co.za or Elsabe on (021) 976-8129 (mornings only) for an invitation. In particular, founding sponsors, editorial board members and reviewers will hopefully attend and the intention is to have at least one prominent editor from an overseas journal present.

The front cover of CVJSA is being redesigned for added impact and a more modern appearance. The present website is also to be redesigned for the CVJA to make it more user friendly and functional. The sister publication, the *South African Journal for Diabetes and Vascular Disease* will move to a new website.

As we face the future, we are aware of the economic stresses worldwide and must consider strategic ways to overcome this period of recession, which is expected to be of considerable duration.

Smaller projects of significance that we have undertaken to increase income include attendance at international congresses for the advance electronic reporting of international trial results, and the publication of the *Pocket Guide to the Treatment of Heart Failure in a Multicultural Society* (electronic and printed versions), which will be updated on an ongoing basis. We will consider the production of other guides on cardiovascular medicine in the future.

All in all, we have had a challenging but productive 20 years and thank our many supporters who have encouraged us to reach the highest standard possible.

Season’s greetings. We wish you and yours a happy and prosperous new year.

